# An unusual cause of collapsed lung after transhiatal oesophagectomy: a case report

**DOI:** 10.1186/1757-1626-1-55

**Published:** 2008-07-23

**Authors:** GKJ Guthrie, LH Moyes, MJ Forshaw

**Affiliations:** 1 Regional Oesophagogastric Unit, Royal Infirmary, 84 Castle Street, Glasgow, G4 0SF, UK

## Abstract

Transhiatal oesophagectomy is an established technique for resection of tumours of the lower oesophagus and oesophagogastric junction. The authors describe a previously unreported serious complication associated with placement of a corrugated neck drain during transhiatal oesophagectomy. A 63 year old man was admitted for transhiatal oesophagectomy for resection of a lower third oesophageal tumour. Post operatively he developed a left sided pneumothorax which did not improve despite numerous chest drains. The subcutaneous corrugated neck drain was removed with immediate inflation of the lung. We report an important potential complication that surgeons in several specialties should be aware of, especially in the use of corrugated neck drains following transhiatal oesophagectomy.

## Background

Transhiatal oesophagectomy is an established technique for resection of tumours of the lower oesophageus and oesophagogastric junction [1]. Many surgeons choose this approach in preference to a two stage approach (Ivor Lewis oesophagectomy) as the potential morbidity from a thoracotomy and the lesser consequences of an anastomotic leak from a cervical anastomosis are avoided. It has been our practice to place a corrugated drain in the left cervical wound following oesophagectomy to encourage any anastomotic leakage towards the skin [2,3].

The authors describe a serious complication associated with placement of a corrugated neck drain during transhiatal oesophagectomy.

## Case report

A 63 year old previously healthy man presented with reflux and dysphagia. Endoscopy revealed a stricturing lower third adenocarcinoma. Staging by means of computed tomography and endoscopic ultrasound showed no metastases and the tumour was staged as T3, N1, M0. He received two cycles of neoadjuvant chemotherapy.

**Figure 1:**
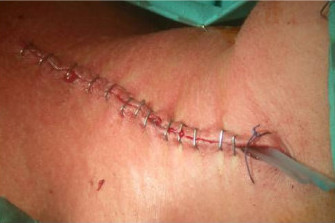
Photograph showing a subcutaneous neck drain in the left neck wound.

At transhiatal oesophagectomy, a bulky tumour just above the diaphragmatic hiatus was identified. To obtain clear circumferential margins, the mediastinal pleura bilaterally sides was excised en bloc with the tumour. Two transhiatal chest drains were inserted through the diaphragmatic hiatus into each pleural cavity. As per our standard practice, a corrugated drain (Portex corrugated drain, Smiths Medical, USA) was placed within the left cervical wound (Figure 1).

**Figure 2:**
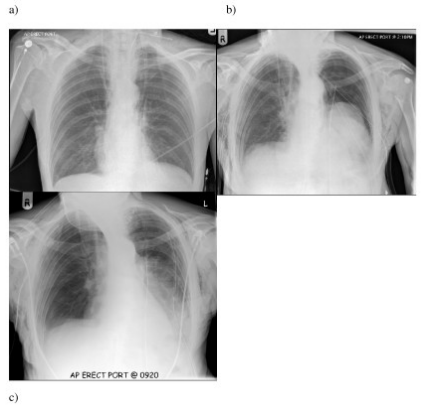
**Series of chest radiographs**. a) Chest radiograph in recovery showing bilateral pneumothoraces despite transhiatal chest drains. Note corrugated drain in left side of neck. b) Chest radiograph taken on day 1 revealing collapse of left lung despite chest drainage. Note resolution of right pneumothorax. c) Chest radiograph demonstrating full expansion of lung after removal of corrugated neck drain.

Postoperatively, both chest drains were noted to be bubbling significantly. A chest radiograph revealed bilateral 30% pneumothoraces (Figure 2a) requiring the insertion of two further trans-thoracic chest drains. A subsequent chest radiograph revealed near complete resolution of the pneumothoraces. The next day, he was breathless with signs of respiratory distress. A repeat chest radiograph revealed that the left lung had now become totally collapsed (Figure 2b). The working diagnosis at this stage was either an air leak from the lung parenchyma or less likely, a tracheobronchial injury. A third chest drain was inserted on the left side but the left lung still remained collapsed. At this point, the patient developed worsening respiratory distress and a flexible bronchoscopy under general anaesthetic was arranged. Under general anaesthesia, the air leak from the chest drains immediately ceased following the insertion of a cuffed endotracheal tube. No tracheobronchial injury was identified.

At this point, the possibility of air ingress through the neck drain was considered. The drain was removed and the skin closed with staples. His left lung fully re-inflated after removal of the neck drain within a few hours (Figure 2c). He made an otherwise uneventful recovery and was discharged home 17 days after his operation.

## Discussion

This case highlights the potential danger associated with the use of corrugated neck drains after transhiatal oesophagectomy. It is acknowledged that slipped chest drains can cause postoperative air leaks but there are no reports in the literature of pneumothoraces associated with subcutaneous neck drains. Our senior author's experience and a larger series of over 2000 oesophagectomies have not encountered this complication [1,4].

Our case suggests that the corrugated neck drain caused a continuous air leak, resulting in pathophysiology mimicking a sucking chest wound. A sucking pneumothorax occurs when the connection between the pleural space and the atmosphere remains patent. With large defects, there is less resistance to airflow into the pleural space than into the lung itself, resulting in reduced air entry into the lung.

We are unclear why this patient developed a pneumothorax when other transhiatal oesophagectomies performed in a similar fashion did not. Presumably in other cases when pleura is excised, the gastric pull-up tube fills the defect. We speculate that the gastric tube may have eventually sealed the right pleural defect but the left side remained collapsed because of a continued air leak. During general anaesthesia the presence of a cuffed endotracheal tube prevented further air entry through the drain. This may have occurred due to either the local pressure effect of the endotracheal tube narrowing the thoracic inlet, or due to the positive pressure exerted by ventilation resulting in higher intrathoracic pressure thus preventing further air ingress.

The differential diagnoses of a collapsed lung post oesophagectomy were considered throughout the assessment of our patient. Potential causes include tracheobronchial injury, parenchymal air leak or slipped chest drains. Persistent air leak through a chest drain or surgical emphysema of the face and neck in the early post operative period is indicative of airway injury [5]. Bronchoscopy will reveal the site of injury. If the lung is collapsed and does not expand on applying negative suction to the chest drain, tracheobronchial injury should be suspected and managed by primary closure of the defect [4]. Parenchymal air leaks tend to increase with positive pressure ventilation so this diagnosis was less likely in our patient as the air leakage improved with intubation and positive pressure ventilation. Slippage of chest drains in the post operative period is a recognised problem but this is normally apparent clinically with visible fenestrations outside the thorax, this was not the case with our patient.

## Conclusion

This experience has highlighted the potential danger of placement of corrugated neck drains during transhiatal oesophagectomy. We suggest that if a subcutaneous drain is necessary, a sealed drain or a sealed occlusive dressing over a corrugated drain should be used to avoid this injury.

## Consent

Written informed consent was obtained from the patient for publication of this case report and accompanying images. A copy of the written consent is available for review by the Editor-in-Chief of this journal.

## Competing interests

The authors declare that they have no competing interests.

## Authors' contributions

GG and LHM reviewed the case notes and participated in writing the manuscript. MJF read and approved the final manuscript.
